# *FCGR2/3* polymorphisms are associated with susceptibility to Kawasaki disease but do not predict intravenous immunoglobulin resistance and coronary artery aneurysms

**DOI:** 10.3389/fimmu.2024.1323171

**Published:** 2024-09-18

**Authors:** Paula Uittenbogaard, Stejara A. Netea, Michael W. T. Tanck, Judy Geissler, Piotr Buda, Monika Kowalczyk-Domagała, Magdalena Okarska-Napierała, Diana van Stijn, Carline E. Tacke, US Kawasaki Disease Genetics Consortium, David P. Burgner, Chisato Shimizu, Jane C. Burns, Irene M. Kuipers, Taco W. Kuijpers, Sietse Q. Nagelkerke

**Affiliations:** ^1^ Department of Blood Cell Research, Sanquin Research Institute, University of Amsterdam (UvA), Amsterdam, Netherlands; ^2^ Department of Pediatric Immunology, Rheumatology and Infectious Diseases, Emma Children’s Hospital, Amsterdam University Medical Center (Amsterdam UMC), UvA, Amsterdam, Netherlands; ^3^ Department of Experimental Immunology, Amsterdam Institute for Infection and Immunity, Amsterdam UMC, UvA, Amsterdam, Netherlands; ^4^ Department of Epidemiology and Data Science, Amsterdam UMC, Location UvA, Amsterdam, Netherlands; ^5^ Department of Pediatrics, Nutrition and Metabolic Diseases, The Children’s Memorial Health Institute, Warsaw, Poland; ^6^ Department of Pediatric Cardiology, The Children’s Memorial Health Institute, Warsaw, Poland; ^7^ Department of Pediatrics with Clinical Assessment Unit, Medical University of Warsaw, Warsaw, Poland; ^8^ Murdoch Children’s Research Institute, Royal Children’s Hospital, Melbourne, VIC, Australia; ^9^ Department of Pediatrics, University of Melbourne, Melbourne, VIC, Australia; ^10^ Department of Pediatrics, University of California, San Diego, La Jolla, CA, United States; ^11^ Department of Pediatric Cardiology, Amsterdam UMC, UvA, Amsterdam, Netherlands

**Keywords:** Kawasaki disease, IVIg, CAA, FCGR2, FCGR3, genetics, FCGR2Ap.His166Arg

## Abstract

**Introduction:**

Kawasaki disease (KD) is a pediatric vasculitis that can result in coronary artery aneurysm (CAA) formation, which is a dangerous complication. Treatment with intravenous immunoglobulin (IVIg) significantly decreases the risk of CAA, possibly through competitive binding to Fc-gamma receptors (Fc*γ*Rs), which reduces the binding of pathological immune complexes. However, ~20% of children have recrudescence of fever and have an increased risk of CAA. Therefore, we aimed to identify genetic markers at the *FCGR2/3* locus associated with susceptibility to KD, IVIg resistance, or CAA.

**Materials and methods:**

We investigated the association of single-nucleotide polymorphisms (SNPs) and copy number variations (CNVs) at the *FCGR2/3* locus with KD susceptibility, IVIg resistance, and CAA risk using a family-based test (KD susceptibility) and case–control analyses (IVIg resistance and CAA risk) in different cohorts, adding up to a total of 1,167 KD cases. We performed a meta-analysis on IVIg resistance and CAA risk including all cohorts supplemented by previous studies identified through a systematic search.

**Results:**

*FCGR2A-*p.166His was confirmed to be strongly associated with KD susceptibility (Z = 3.17, *p* = 0.0015). In case–control analyses, all of the investigated genetic variations at the *FCGR2/3* locus were generally not associated with IVIg resistance or with CAA risk, apart from a possible association in a Polish cohort for the *FCGR3B-*NA2 haplotype (OR = 2.15, 95% CI = 1.15–4.01, *p* = 0.02). Meta-analyses of all available cohorts revealed no significant associations of the *FCGR2/3* locus with IVIg resistance or CAA risk.

**Discussion:**

**FCGR2/3 polymorphisms are associated with susceptibility to KD but not with IVIg resistance and CAA formation.** Currently known genetic variations at the *FCGR2/3* locus are not useful in prediction models for IVIg resistance or CAA risk.

## Introduction

Kawasaki disease (KD) is a pediatric vasculitis involving the coronary arteries that mainly affects children under the age of 5 (1). The disease can result in coronary artery aneurysm (CAA) formation, which occurs in up to 25% of untreated cases, making it the most common cause of acquired heart disease in children in developed countries (2). The risk of CAA has significantly diminished since the introduction of treatment with human-pooled intravenous immunoglobulin (IVIg) (3). Although its exact mechanism of action remains uncertain, various mechanisms of action have been proposed (4–7). Similarly, there is the involvement of the Fc-gamma receptors (Fc*γ*Rs), for instance, by competitive binding of IVIg to the Fc*γ*Rs, thereby reducing the binding of pathological immune complexes and inhibiting cellular activation. In turn, IVIg-resistant patients have an increased risk of developing CAA (8, 9). IVIg resistance occurs in approximately 20% of treated patients (10), with up to 20% of affected children developing CAA despite timely treatment (9). This underlines the need to better understand the mechanisms behind poor treatment response and the importance of identifying prognostic markers that accurately predict children at risk for poor treatment response or of developing CAA. Although risk scores have been developed to predict IVIg resistance including various laboratory findings (11–14), these risk score systems have been developed for the Japanese population and have a limited predictive value in non-Japanese populations (15, 16). As has also been proposed in the KD guidelines of the American Heart Association (AHA) (17) and the European Initiative Single Hub and Access point for pediatric Rheumatology in Europe (SHARE) (18), better predictive models, possibly incorporating biomarkers or genetic variants, are needed for non-Asian populations.

The exact etiology of KD is unknown, but it is hypothesized to be a post-infectious hyperinflammatory reaction in children with a genetic susceptibility. The hypothesis of a post-infectious etiology was reinforced by the recent outbreak of multisystem inflammatory syndrome in children, which shares some clinical features with KD and is triggered by severe acute respiratory syndrome coronavirus 2 (SARS-CoV-2) infection (19, 20). Several findings point to a genetic susceptibility for KD. Firstly, the incidence of KD differs widely per country, with the incidence in European countries ranging between 10 and 15 per 100,000 children under the age of 5 per year (21). Over 10-fold higher incidence rates of KD have been reported in Asian countries, particularly in Japanese and Koreans, which persist when patients of these ethnicities migrate to other countries (22, 23). Secondly, as has been shown by twin studies and familial cases, siblings of patients with KD have a higher KD risk compared with the general population (24, 25).

Among other gene regions (encoding, e.g., caspase 3, human leukocyte antigen class II, B-cell lymphoid kinase, inositol 1,4,5-trisphosphate kinase C, and CD40 protein) (17), KD has been linked to the gene cluster of the Fc*γ*R family (26). This *FCGR2/3* locus consists of genes encoding the five low- to medium-affinity Fc*γ*Rs (*FCGR2A*, *FCGR2B*, *FCGR2C*, *FCGR3A*, and *FCGR3B*) that either activate or inhibit (*FCGR2B*) cellular immune functions after binding immunoglobulin G (IgG). Various single-nucleotide polymorphisms (SNPs), haplotypes, and copy number variations (CNVs) at the *FCGR2/3* locus impact receptor function and expression levels (27–29).

Most studies on the association of KD with the *FCGR2/3* locus have focused on the genetic association of the SNP *FCGR2A-*p.His166Arg (rs1801274, formerly known as *FCGR2A-*p.His131Arg) with KD susceptibility and have shown that *FCGR2A-*p.166His is a marker of increased susceptibility (30–33). This SNP in the *FCGR2A* gene can be typed with conventional methods, such as genome-wide association study (GWAS). However, the *FCGR2/3* locus is notoriously difficult to genotype. Firstly, it comprises a large segmental duplication with over 98% homology shared between the duplicated regions (30). Secondly, CNV is common in this locus, resulting in a highly polymorphic region. There is a strong linkage disequilibrium (LD) between various variants (27). Most of the known variants on the *FCGR2/3* locus cannot be included in GWAS analyses.

Comprehensive analysis of the *FCGR2/3* locus can be achieved using the previously described multiplex ligation-dependent probe amplification (MLPA), which can reliably distinguish the currently known SNPs, haplotypes, and CNVs. These SNPs include *FCGR2A-*p.Gln62Trp, *FCGR2A-*p.His166Arg, *FCGR2B-*p.Ile232Thr, and *FCGR3A-*p.Val176Phe. Known haplotypes that can be distinguished include the *FCGR2B* and *FCGR2C* promoter haplotypes 2B.1/2B.2/2B.4, *FCGR3B* NA1/NA2/SH haplotypes (which encode the human neutrophil antigen), and *FCGR2C* Stop/ORF/non-classic ORF haplotypes. CNVs (both deletions and duplications) occur in the CNR1–CNR4 regions, generally involving either *FCGR3A-FCGR2C* or *FCGR2C-FCGR3B*, and can be distinguished. Using MLPA, Nagelkerke et al. found that, in addition to *FCGR2A-*p.His166Arg, the *FCGR2C-*ORF haplotype (rs759550223 and rs7677413) was significantly associated with susceptibility to KD in the European population (30). Although the association of *FCGR2A-*p.His166Arg appears to be independent of ethnicity, a clear ethnic variation was seen for the *FCGR2C-*ORF. This haplotype was associated with susceptibility to KD in Europeans but is virtually absent in Asian subjects, stressing the importance of accounting for ethnicity when investigating genetic associations in KD.

The association of *FCGR2A-*p.His166Arg with KD susceptibility raises the question of whether this SNP or other genetic variants at the *FCGR2/3* locus may also impact the clinical outcomes of patients with KD, particularly the risk of IVIg resistance and CAA. The few studies that investigated such associations lack consensus and often only focus on one or several Fc*γ*Rs in relatively small patient populations (31–36). This highlights the need to further fine-map genetic associations at the *FCGR2/3* locus accounting for ethnic variations in extensive patient populations. In the present study, we, therefore, investigated the association of all currently known functionally relevant SNPs, haplotypes, and CNVs at the *FCGR2/3* locus with susceptibility to KD, response to treatment, and risk of CAA development using a family-based test (KD susceptibility) and case–control analyses (CAA and IVIg response). Cases were recruited from four cohorts from different parts of Europe (the Netherlands and Poland), the United States, and Australia. Subsequently, we performed a meta-analysis for the association of *FCGR2/3* genetic variations with IVIg resistance and CAA risk in all four cohorts, supplemented by studies identified through a systematic review.

## Materials and methods

### Power analysis

We used the Quanto software to calculate the statistical power of our cohort size (37). We expected a total cohort of at least 800 subjects (children with KD) with an estimated prevalence of IVIg resistance of 0.22 and an estimated CAA prevalence of 0.30. We calculated the power to detect an odds ratio of 2 per minor allele for variants with minor allele frequencies (MAFs) ranging from 0.05 to 0.5, assuming an allelic genetic model and setting the significance threshold (*p*-value) at 0.05. For IVIg, the power to detect an OR of 2 was >80% for variants with an MAF of 0.05 and >90% for an MAF ≥0.1. For CAA, the power was >90% for the whole MAF range.

### Subjects

#### KD cases

Complete KD cases, who had not received prior immunomodulatory or immunosuppressive treatment, were recruited from the Netherlands (*n* = 388), the United States (*n* = 447), Australia (*n* = 213), and Poland (*n* = 119) with diagnosis based on the standard diagnostic clinical criteria from the AHA (17). In total, 1,167 patients were included in the study. A total of 762 patients (701 complete trios and 61 incomplete trios) were included in the transmission disequilibrium test (TDT) analysis for susceptibility to KD.

This TDT analysis is an extension of our previously published TDT analysis (30) using the same trios supplemented by an additional 155 trios from the Netherlands.

As per the 2017 AHA guidelines, primary treatment consisted of a single IVIg infusion (2 g/kg) and aspirin (started at 30–50 mg kg^−1^ day^−1^ and reduced to 3–5 mg kg^−1^ day^−1^ once fever subsided for 48 h) (17).

For associations within the patient groups for susceptibility to CAA and response to IVIg treatment, we performed case–control analyses for all patients for which these clinical parameters, as well as self-reported ethnicity, were documented. For both these case–control analyses, there was considerable, but not complete, overlap with the patients in the TDT. Many of the KD cases were previously studied for general susceptibility to KD in our GWAS (33) and follow-up study (27). There was no overlap with the patients with KD described by Biezeveld et al. (28). In total, 872 patients of self-reported European descent and 60 patients of self-reported Asian descent were included in the case–control analyses. For an overview of the cohorts, see **Supplementary Table 1**.

### Data collection

#### Clinical data

Clinical information from the disease episode (i.e., sex, ethnicity, treatment response, and CAA development) was collected using electronic health records and processed in a combined database (REDCap and Castor). CAAs were defined as a coronary artery with a *Z*-score ≥2.5 (17). In the case–control analysis, cases were defined as patients with CAA, whereas controls were defined as patients without CAA. IVIg resistance was defined as persistent or recurrent fever >38°C at >36 h and <7 days after completing the initial IVIg infusion in patients treated with IVIg ≤10 days post-onset of fever (17). In this case–control analysis, cases were defined as patients having an adequate IVIg response, while controls were defined as patients with IVIg non-responsiveness. This case–control analysis only included patients treated with IVIg ≤10 days of the onset of the disease.

#### MLPA

Genomic DNA from whole blood was isolated using the QIAamp Blood Mini kit (Qiagen, Hilden, Germany). Genomic DNA from saliva was isolated using the Oragene DNA self-collection kit (DNA Genotek, Ontario, Canada) or the Puregene DNA purification kit (Qiagen, Hilden, Germany).

SNPs and CNVs in the low-affinity *FCGR* genes were determined using an *FCGR-*specific MLPA assay as described previously (30, 38) (P110 and P111, MRC-Holland, Amsterdam, the Netherlands). The MLPA contained probes to detect all the currently known SNPs and the gene-specific probes to determine CNVs. DNA samples of three healthy individuals well typed for all CNVs and SNPs were included as a reference in all experiments. The results were analyzed using the program Genemarker version 3.0.0 (Soft Genetics LLC, State College, PA, USA), with consideration for the homology within the locus and taking into account known LD aiding in the construction of haplotypes, as described previously (29, 30).

#### Family-based analyses

For the family-based analysis using FBAT (see *Statistical analysis*), phased genotypes were required (i.e., separated into the paternal and maternal alleles for each individual). Because the MLPA results could not be phased over the full locus, we separated the genotypes for each SNP manually, assuming an “even distribution” of the copies over the columns. For example, if an individual had two allele copies (no CNV), then we assumed that each allele contained one copy unless our trio data suggested otherwise (rare occasions). Regarding CNVs of *FCGR3B* and the NA1 and NA2(SH) genotypes, previous evidence has shown that the NA1 and NA2(SH) genotypes are generally on the same allele (the duplication allele) (39). For the other genotypes, this was not found, and we assumed that the duplication allele contained the same genotypes (e.g., *FCGR2C*: ORF-ORF and Stop-Stop). In case of ambiguity, the genotypes were solved by using the trio information combined with the linkage information on the locus.

### Statistical analysis

Firstly, we performed a (multi-marker) TDT (using an FBAT toolkit in R) in the parent-affected offspring trios to investigate the association between KD susceptibility and the various SNPs, CNVs, and haplotypes.

Thereafter, differences in the prevalence of variants were compared between the cohorts using Fisher’s exact test to assess which data could be pooled in further analyses. There were significant differences in the variants for one or more variants between all groups, except for the US and Australian cohorts. The latter cohorts showed no significant differences and were merged into a single cohort (data not shown).

Subsequently, case–control analyses of the association of each genetic variant (allelic model) with IVIg resistance and CAA development were conducted. Analyses were performed in R using one-sided Fisher’s exact tests and univariable logistic regression models. Variants that were significantly associated with IVIg resistance or CAA development in previous literature reports and/or in our own analyses were included in a meta-analysis of the findings found in all separate cohorts (meta package in R). To assess differences by ethnicity, we primarily separated the ethnic groups in the case–control analysis and then performed a meta-analysis with the complete dataset.

A *p*-value below 0.05 was considered statistically significant. Statistical analyses were performed in R version 4.1.

### Systematic literature review

A systematic search in PubMed/Medline was conducted on March 2, 2023, to identify all studies that reported the relationship between SNPs in the *FCGR2/3* locus and IVIg resistance and CAAs in patients with KD. The search strategy included the following search terms: (FCGR* OR FC*γ*R* OR Fc-gamma OR CD64 OR CD32 OR CD16 OR rs1801274 OR rs759550223 OR rs76277413 OR rs149754834 OR rs201218628 OR rs143796418 OR rs396991 OR rs5030738 OR rs1050501) AND (“Mucocutaneous Lymph Node Syndrome” [Mesh] OR “Mucocutaneous Lymph Node Syndrome” OR MCLS OR “Kawasaki disease” OR KD OR “Kawasaki-disease” OR “Kawasaki Syndrome” OR “Acute inflammatory Vasculitis”). Studies solely reporting on DNA methylation status were excluded. We extracted data on the study characteristics (e.g., study design, number of patients enrolled in the study, number of patients fulfilling the review’s inclusion criteria, ethnicity of the patients, length of follow-up, and methods), outcome measures (e.g., definitions used and detection methods), and the main study results. A meta-analysis was performed with the pooled data of comparable studies with similar definitions (KD, IVIg resistance, and CAA). We collected all available allele-specific beta coefficients and standard errors (calculated using the odds ratios and 95% confidence intervals) from univariable logistic regressions performed to investigate the association between various allele frequencies within the Fc*γ*R locus and the risk of IVIg resistance and/or CAA development. When only the genotypic odds ratios were reported, then we calculated the allele-specific odds ratios by using the genotype frequencies. Lastly, we conducted a meta-analysis that included our own data and the data extracted from the studies identified in the systematic literature review. The meta-analysis was performed using the meta package (version 6.2-0) in R. Forest plots were made in R using the forest package.

### Study approval

Informed consent and written approval were obtained. All patients were included in accordance with the study protocol, the International Conference on Harmonization Good clinical Practice guidelines, and the provisions of the Declaration of Helsinki. Further information per cohort is listed below.

#### The Netherlands

Subjects from the Netherlands were recruited at the Amsterdam UMC and participating hospitals in the broad Amsterdam region as part of the long-term observational Kawasaki disease study as approved by the Medical Ethical Board of the AMC, with the reference number 2012_155 (no. NL41023.018.12).

#### The United States

Subjects from the United States were recruited at Rady Children’s Hospital San Diego (*n* = 241), Boston Children’s Hospital (*n* = 81), Northwestern University (*n* = 37), University of Hawaii (*n* = 19), Children’s Hospital of Los Angeles (*n* = 6), and individuals across the US who directly contacted the University of California San Diego to participate (*n* = 63). The Human Research Protection Program of the University of California San Diego approved this research protocol (IRB 170790). All parents and subjects gave written consent or assent as appropriate.

#### Australia

Subjects from Australia (*n* = 213) were recruited in accordance with the protocol approved by the ethics committees of all participating tertiary pediatric hospitals.

#### Poland

The blood samples of 119 patients diagnosed with KD were collected between 2016 and 2020, isolated, and stored at the Department of Pediatrics, Nutrition and Metabolic Diseases, Children’s Memorial Health Institute in Warsaw, Poland. All children were born in Poland (Mazowieckie Voivodeship) and were of European ethnic descent. Patients were included as part of the project “Searching for molecular markers related to Kawasaki disease,” which has been approved by the Bioethics Committee at the Children’s Memorial Health Institute Review Board.

## Results

In total, 2,631 individuals were genotyped. A detailed description of the included patients is shown in **Supplementary Table 1**.

### Association of *FCGR2A*-p.His166Arg SNP with KD susceptibility

In our TDT analysis (FBAT), we included 762 families, with 701 complete trios and 61 incomplete trios. A significant association with susceptibility to KD of the *FCGR2A-*p.His166Arg SNP, which has been previously confirmed in meta-analyses, was found in our cohort in the single-marker FBAT analysis (**Supplementary Table 2**) and was also confirmed in the multi-marker FBAT (**Table 1**). Neither *FCGR2C-*ORF nor any of the other SNPs were significantly associated with susceptibility to KD (**Table 1**).

**Table 1:** Multi-marker transmission disequilibrium test for the different variants at the *FCGR2/3* locus in a family-based association study.

SNP/haplotype	No. of alleles^a^	No. of families^b^	Degree of freedom	Chi-square	*p*-value
*FCGR2A* p.His166Arg	2	515	1	10.05	**0.0015**
*FCGR2A* p.Gln62Trp	2	261	1	0.17	0.68
*FCGR2B* p.Ile232Thr	2	262	1	0.05	0.82
*FCGR3A* p.Val176Phe	7	511	6	7.70	0.26
*FCGR2B* promoter	3	242	2	4.72	0.09
*FCGR2C* promoter	11	450	5	5.18	0.39
*FCGR3B* NA1/NA2/SH	12	546	7	6.26	0.51
*FCGR2C* ORF/NC ORF/Stop	12	462	6	7.62	0.27

NC ORF, non-classic ORF.

a Number of different alleles in the cohort. For genes without copy number variation, this is 2; for genes with copy number variations, this can be higher, for example when there is a null allele due to a deletion or an allele with two variants on one allele due to duplication.

b Number of informative families in which ≥1 parent is heterozygous for the indicated allele or haplotype. Bold values are statistically significant (P<0.05).

### Association of the *FCGR2/3* SNPs and haplotypes with IVIg resistance in the case–control analysis and meta-analysis

We performed a case–control analysis including KD patients with adequate IVIg response as cases and patients with IVIg non-responsiveness as controls. None of the variants tested were associated with IVIg response in our case–control analysis for the combined US and Australian cohort (**Supplementary Table 3**), the Dutch cohort (**Supplementary Table 4**), or the Polish cohort (**Supplementary Table 5**). The subsequent meta-analysis of our different cohorts did not reveal associations either (**Supplementary Figure 1**).

**Figure 1:**
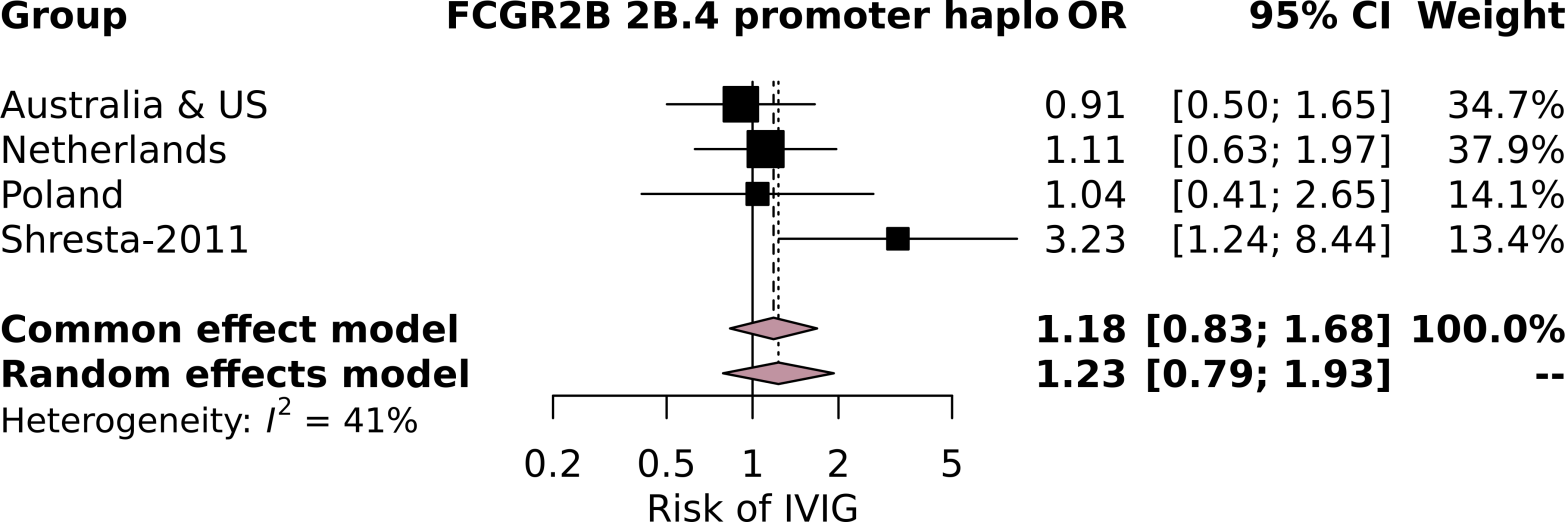
Forest plot of the meta-analysis investigating the association of the *FCGR2B* 2B.4 promoter haplotype, which contains the *FCGR2B*-c.-120T/A minor allele A, investigated in Shresta-2011, with intravenous immunoglobulin (IVIg) resistance in individuals of European descent in the current study cohorts and the cohort from Shresta-2011.

Subsequently, we performed an additional meta-analysis based on studies identified through a systematic search (**Supplementary Figure 1**). Although four studies focused on the relationship between variants at the *FCGR2/3* locus with IVIg resistance (31, 34, 35, 40), only a single study (34) could be included in our subsequent meta-analysis (**Supplementary Table 6**; **Supplementary Figure 2**). This study focused on SNPs in *FCGR2B*, including the SNP *FCGR2B-*p.Ile232Thr, which was not significantly associated with IVIg resistance in this study, nor in our meta-analysis. The study also reported that minor allele A at *FCGR2B*-c.-120T/A was more frequently detected in patients of European descent who responded to IVIg (OR = 3.23, 95% CI = 1.22–8.33, *p* = 0.01) (34). We also included the latter SNP in our meta-analysis as part of the *FCGR2B* promoter haplotype 2B.4 and could not reproduce the significant association with IVIg (**Figure 1**). While this SNP can also occur before *FCGR2C*, this is extremely rare [5/2,631 (0.19%) samples in the current study] and likely does not affect our analysis. The other studies could not be included in our meta-analysis because they 1) compared patients with IVIg resistance to healthy controls (31) and 2) did not report allelic frequencies (35, 40).

Although not included in the meta-analysis, one of these studies found over-transmission of *FCGR3B-*NA1 among IVIg non-responders (cases) compared to healthy controls (controls) (31). In another study, the copy numbers of *FCGR2C* in Hispanics and *FCGR3B* in the European population were found to be associated with IVIg resistance (35). No significant associations of *FCGR2A-*p.166His with IVIg resistance were found (31, 40).

### Association of the *FCGR2/3* SNPs and haplotypes with CAA risk in the case–control analysis

We also performed a case–control analysis including KD patients with CAAs as cases and patients without CAAs as controls. On the basis of self-reported ethnicity, we investigated informative cases of European descent (the Netherlands: 65 cases, 291 controls; Poland: 43 cases, 76 controls; and Australia and USA: 105 cases, 262 controls) and Asian ethnicity (Australia and USA: 23 cases, 46 controls) separately. The various genotypes and allele frequencies of the CNVs and SNPs are shown in **Supplementary Tables 7–9**.

In the Polish cohort, the *FCGR3B-*NA2 haplotype was significantly associated with CAA risk (Fisher’s exact test: *p* = 0.05; single logistic regression: *p* = 0.02; OR = 2.15, 95% CI = 1.15–4.01). In the same cohort, having less than two copies of CNR1 was also associated with CAA risk using Fisher’s exact test (*p* = 0.05), but was not confirmed in the single logistic regression (OR = 0, 95% CI = 0.00–Inf, *p* = 0.99), presumably because of the low MAF of less than two copies (deletion) of this CNR.

None of the other SNPs or haplotypes were associated with CAA risk in any of the cohorts investigated (**Supplementary Tables 7–9**).

### Association of the *FCGR2/3* SNPs or haplotypes with CAA risk in the meta-analysis

In our systematic literature review, we identified studies that focused on the relationship between variants at the *FCGR2/3* locus and the risk of CAA (28, 31, 32, 36, 40, 41) (**Supplementary Table 6**; **Supplementary Figure 2**). Four of these focused on the association of *FCGR2A-*p.His166Arg with clinical outcomes. *FCGR2A-*p.166His was significantly associated with CAA risk in one (Taniuchi et al.) of the four studies (28, 32, 36, 41) in which it was investigated. In addition, over-transmission of *FCGR3B-*NA1 was described in one study among patients with CAAs (31) but was not confirmed in another study (32). None of the other variants investigated were associated with CAA risk.

**Figure 2:**
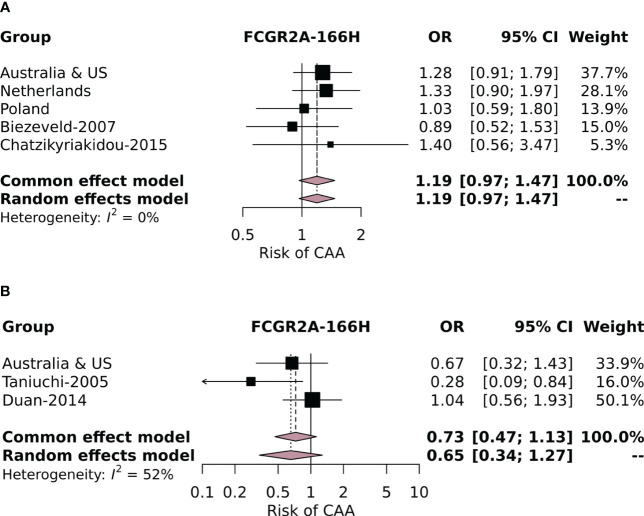
Forest plot of the meta-analysis investigating the association of the *FCGR2A*-p.His166Arg SNP with coronary artery aneurysm (CAA) risk in individuals of European descent in the current study cohorts and cohorts identified in the literature review **(A)** and in **(B)** Asian individuals from the Australian and US cohorts and the Asian cohorts identified in the literature review.

Subsequently, we performed a meta-analysis including our own cohorts and the four studies that used similar definitions for cases (KD patients with CAAs) and controls (KD patients without CAAs). The variants investigated in the meta-analysis including the studies from the literature were *FCGR2A-*p.His166Arg, *FCGR2B-*p.Ile232Thr, *FCGR3A-*p.Phe176Val, and *FCGR3B-*NA1. None of the variants investigated were associated with CAA risk in our meta-analysis of the total population (**Supplementary Figure 3**). This included the *FCGR3B-*NA2 haplotype, which, although significantly associated in the current Polish cohort, was not associated in the meta-analysis. Finally, because a strong correlation of *FCGR2A-*p.166His with CAA risk was reported by Taniuchi et al. (32), we also performed a separate meta-analysis with the European (from all four current study cohorts) (28, 41) and Asian subpopulations (from the current Australian and US cohorts) (32, 36) to exclude an ethnicity-dependent association. No significant effect of *FCGR2A-*p.166His on CAA risk was found in either the European or the Asian subpopulation (**Figures 2A, B**, respectively).

## Discussion

In the current study, we investigated the association of all currently known functionally relevant SNPs and CNVs at the *FCGR2/3* locus with KD susceptibility, IVIg resistance, and CAA risk in an extensive cohort, taking into account ethnic differences and using a meta-analysis. We confirmed the established association of the *FCGR2A-*p.His166Arg SNP with susceptibility to KD, whereas none of the investigated genetic variations at the *FCGR2/3* locus were associated with resistance to IVIg or risk of CAA.

Firstly, we reproduced the association of the *FCGR2A-*p.His166Arg SNP with susceptibility to KD in an extended single- and multi-marker TDT analysis, which corrected for ethnic differences (parents of the affected child were used as internal controls). This overall association reinforces the findings in our initial GWAS in KD (33) and the previous meta-analyses (42–44). The SNP results in an arginine (R) or histidine (H) at amino position 131 in the mature protein after cleaving of signal peptides, with the histidine variant being a risk factor for KD. It is important to note that Fc*γ*RIIa-His131 can bind human IgG2, whereas Fc*γ*RIIa-Arg131 cannot (45, 46). IgG2 is the second most prevalent IgG in the IVIg preparations; the distribution of the different IgG subclasses in IVIg varies slightly per preparation, and an IVIg infusion contains approximately 54%–70% IgG1, 29%–45% IgG2, 1%–4% IgG3, and 0%–0.5% IgG4 (47).

It has been previously suggested that the genetic association may only account for susceptibility to KD in Asian patients but not in European patients (43). A more recent meta-analysis (42) did find a significant association of the SNP with KD susceptibility in sub-analyses including solely patients of European descent, although methodological issues of the study resulted in the inclusion of two studies with the same study population (27, 33), which may have resulted in bias. Nevertheless, this study did not include the data of the TDT analysis that we have previously performed for *FCGR2A-*p.His166Arg in European patients only (48). In this cohort, the *FCGR2A*-p.166His was also significantly associated with susceptibility to KD (Z = 2.94, *p* = 0.003) (19). Therefore, we postulate that the association of the *FCGR2A-* p.His166Arg SNP with KD susceptibility is general and holds true in different ethnicities.

Interestingly, neither the *FCGR2C-*ORF haplotype nor any of the other *FCGR2/3* variants were associated with KD susceptibility in our TDT analyses. This haplotype results in the expression of the activating Fc*γ*RIIc, which is usually not expressed because of a stop codon in exon 3 (38). In our previous study (30), the *FCGR2C-*ORF haplotype was not significantly associated with KD susceptibility in a TDT analysis, but it was associated with KD susceptibility in a case–control analysis in patients with European ancestry and remained significant in a meta-analysis combining the associations of the TDT and case–control analyses. In the current TDT cohort, which largely overlapped with our previous study (30), the SNP was again not significantly associated with KD susceptibility in the TDT analysis, indicating that *FCGR2A* SNP rs1801274 is the major gene variant for the clear association with the disease.

None of the known functional variations at the *FCGR2/3* locus were associated with IVIg resistance or CAA risk in KD in our case–control analyses, apart from the Polish cohort in which the *FCGR3B-*NA2 haplotype was significantly associated with CAA risk. Notably, this cohort also had the highest proportion of patients with IVIg resistance (35% *vs*. 13%–26%), possibly leading to an overestimation of the effect of the *FCGR3B-*NA2 haplotype. In contrast to our own findings, several previous studies have reported associations between various genetic variations and IVIg resistance (31, 34, 35, 40) or CAA risk (28, 31, 32, 36, 40, 41). However, these studies relied on limited study populations [*n* = 177–358 *vs*. *n* = 653 in our cohort (IVIg); *n* = 41–424 per cohort *vs*. *n* = 911 (CAA)], which may have impacted the statistical power and heterogeneity of these studies. On the basis of our combined meta-analysis, we show that a prognostic value of genetic variations at the *FCGR2/3* locus in prediction models for IVIg resistance or CAA risk in KD patients of European descent is unlikely.

The lack of a direct link between polymorphisms in either the activating or inhibitory Fc*γ*Rs indicates that other factors likely play a role. The mechanism of action of IVIg could be explained by additional interactions with multiple other components of the immune system (e.g., dendritic cells, natural killer cells, T cells, and neutralization of autoantibodies) (49). For instance, it has been suggested that IVIg contains antibodies capable of neutralizing cytokines, further strengthened by the beneficial effects of anti-inflammatory treatment with TNF antagonists (50) and IL-1 antagonists (51, 52). Furthermore, it is known that the glycosylation of antibodies significantly impacts their effector functions and immunogenicity (53). Specifically, the proportion of anti-inflammatory sialylated IgG antibodies (up to 15% of healthy serum IgG) (54)) is reduced in inflammatory diseases (55), including KD (56). It has been suggested that α-2,6-sialylated IgG may be the biologically active component for the immunomodulatory effects of IVIg (57, 58), possibly *via* the induction of IL-33 and the increase of the inhibitory FcγRIIb surface expression on macrophages, dendritic cells, and B cells following IVIg infusion (59, 60) although such mechanisms do not occur in all circumstances (61, 62). In the setting of KD, treatment response has previously been shown to only be associated with lower sialylation levels of endogenous IgG, but not therapeutic IVIg (56). In addition, previous studies have indicated distinctive metabolic profiles in IVIg-resistant KD (63, 64) and KD patients with CAAs (65–67), indicating that the mechanisms behind IVIg resistance and CAA formation are multifaceted and require further elucidation.

Although we captured all SNPs and CNVs that are known to date in our current study, future whole-genome sequencing techniques may reveal new functionally relevant variations at the *FCGR2/*3 locus, which could not be captured in the current study.

One of the limitations of our study was that our cohorts mainly included European patients. The underrepresentation of Asian patients, in particular, increases the risk of overlooking ethnicity-specific associations of genetic variations at the *FCGR2/3* locus with clinical outcomes in KD. A second limitation was the low number of studies that could be included in our meta-analysis, partially explained by the rarity of Kawasaki disease, making it challenging to obtain cohorts powered for investigating IVIg resistance and CAA risk. Consequently, we suspect a certain degree of publication bias when no significant effects are identified in (underpowered) patient populations. However, a major strength of our study was our extensive and homogeneous study population, which included only complete KD cases, ensuring sufficient power to investigate the associations with IVIg resistance and CAA risk (see *Materials and methods*). Secondly, we investigated ethnic differences by primarily separating the ethnic groups in the case–control analysis and then performing a meta-analysis including the complete dataset, which indicated minimal heterogeneity (except for the *FCGR3B-*NA2 haplotype, *I* ^2^ = 73%).

In conclusion, our current study does not support the hypothesis that variations within the *FCGR2/3* locus predict resistance to IVIg and risk of CAA (17). Although it is impossible to fully exclude an association of genetic variations at the *FCGR2/3* locus with clinical outcomes in KD, we determined that our patient population offers sufficient statistical power, and our combined meta-analysis indicates that such a correlation is unlikely and that genotyping *FCGR2/3* variants in patients with acute KD to guide initial treatment is not useful.

## Data availability statement

The original contributions presented in the study are publicly available. This data can be found here: https://www.ncbi.nlm.nih.gov/SNP/snp_viewBatch.cgi?sbid=1063642.

## Ethics statement

The studies involving humans were approved by the Medical Ethical Board of the AMC with the reference number 2012_155 (no. NL41023.018.12) Human Research Protection Program of the University of California San Diego (IRB 170790) Bioethics Committee at the Children’s Memorial Health Institute Review Board. The studies were conducted in accordance with the local legislation and institutional requirements. Written informed consent for participation in this study was provided by the participants’ legal guardians/next of kin.

## Author contributions

PU: Data curation, Formal analysis, Investigation, Visualization, Writing – original draft, Writing – review & editing. SN: Data curation, Formal analysis, Investigation, Project administration, Writing – original draft, Writing – review & editing. MT: Formal analysis, Methodology, Supervision, Validation, Writing – review & editing. JG: Data curation, Writing – review & editing. PB: Data curation, Project administration, Writing – review & editing. MK-D: Data curation, Project administration, Writing – review & editing. MO-N: Data curation, Project administration, Writing – review & editing. DvS: Data curation, Project administration, Writing – review & editing. CT: Data curation, Project administration, Writing – review & editing. DB: Data curation, Project administration, Writing – review & editing. CS: Data curation, Project administration, Writing – review & editing. JB: Data curation, Writing – review & editing. IK: Data curation, Project administration, Writing – review & editing. TK: Conceptualization, Data curation, Supervision, Writing – review & editing. SN: Conceptualization, Funding acquisition, Investigation, Methodology, Project administration, Resources, Supervision, Writing – review & editing.
