# Supplementary material for: FCGR2/3 polymorphisms are associated with susceptibility to Kawasaki disease but do not predict intravenous immunoglobulin resistance and coronary artery aneurysms
Source: Front Immunol. 2024 Sep 18;15:1323171. doi: 10.3389/fimmu.2024.1323171 (PMC11445592; doi:10.3389/fimmu.2024.1323171)

***Supplementary material***

FCGR2/3 polymorphisms are associated with susceptibility to Kawasaki disease, but do not predict intravenous immunoglobulin resistance and coronary artery aneurysms

Paula Uittenbogaard^1^†* & Stejara A. Netea^2,3^†, Michael W.T. Tanck^4^, Judy Geissler^1^, Piotr Buda^5^, Monika Kowalczyk-Domagała^6^, Magdalena Okarska-Napierała^7^, Diana van Stijn^2^, Carline E. Tacke^2^, US Kawasaki Disease Genetics Consortium, David P. Burgner^8,9^, Chisato Shimizu^10^, Jane C. Burns^10^, Irene M. Kuipers^11^, Taco W. Kuijpers^1,2^*͛ & Sietse Q. Nagelkerke^1,2^*͛

†These authors contributed equally to this work and share first authorship

*͛These authors contributed equally to this work and share last authorship

**^1^**Department of Blood Cell Research, Sanquin Research Institute, University of Amsterdam (UvA), Amsterdam, The Netherlands

^2^Department of Pediatric Immunology, Rheumatology and Infectious Diseases, Emma Children’s Hospital, Amsterdam University Medical Center (Amsterdam UMC), UvA, Amsterdam, The Netherlands

^3^Department of Experimental Immunology, Amsterdam Institute for Infection & Immunity, Amsterdam UMC, UvA, Amsterdam, The Netherlands

^4^Department of Epidemiology and Data Science, Amsterdam UMC, Location UvA, Amsterdam, The Netherlands

^5^Department of Pediatrics, Nutrition and Metabolic Diseases, The Children’s Memorial Health Institute, Warsaw, Poland

^6^Department of Pediatric Cardiology, The Children’s Memorial Health Institute, Warsaw, Poland

^7^Department of Pediatrics with Clinical Assessment Unit, Medical University of Warsaw, Poland

^8^Murdoch Children’s Research Institute, Royal Children’s Hospital, Melbourne, VIC, Australia

^9^Department of Pediatrics, University of Melbourne, VIC, Australia

^10^Department of Pediatrics, University of California, San Diego, La Jolla, CA, United States

^11^Department of Pediatric Cardiology, Amsterdam UMC, UvA, Amsterdam, The Netherlands

***Correspondence**

Paula Uittenbogaard

[P.Uittenbogaard@sanquin.nl](mailto:P.Uittenbogaard@sanquin.nl)

**Supplementary Table 1**. Overview of individuals that were genotyped in all the cohorts.

| **Clinical parameter** | **United States (n=447), n (%)** | **Australia (n=213), n (%)** | **The Netherlands (n=388), n (%)** | **Poland (n=119), n (%)** |
| --- | --- | --- | --- | --- |
| Male sex | 283 (63%) | 126 (59%) | 231 (60%) | 73 (61%) |
| Median age in years (IQR)  Age <1 year old at onset | 2.87 (1.52-4.83)  66 (17%) |  | 2.98 (1.17-5.00)  86 (21%) | 2.50 (1.06-4.13)  27 (22.5%) |
| *Ethnicity*  European descent  Asian  Other | 214  60  173 | 181  9  23 | 358  7  23 | 119  0  0 |
| *Treatment with IVIg*  Within 10 days  Resistance | 389  94 (24%) | 127  17 (13%) | 291  76 (26%) | 102  36 (35%) |
| *CAA*  Informative cases  CAA | 442  162 (37%) | 182  32 (18%) | 386  73 (19%) | 119  43 (36%) |
| *Trios*  Complete  Incomplete | 348  38 | 104  0 | 249*  4 | 0  0 |
| * This includes one family of 4, with two affected children. | | | | |

**Supplementary Table 2**. Transmission disequilibrium test for the different variants at the *FCGR2/3* locus in a family-based association study for KD susceptibility.

| **Variant (on 1 chromosome)** | **Allele frequency** | **# families*** | **Z** | ***P* value** |
| --- | --- | --- | --- | --- |
| *FCGR2A* p.His166Arg  H  R | 0.577  0.423 | 515  515 | 3.17  -3.17 | 0.0015  0.0015 |
| *FCGR2A* p.Gln62Trp  Q  W | 0.887  0.113 | 261  261 | -0.415  0.415 | 0.678  0.678 |
| *FCGR2B* p.Ile232Thr  I  T | 0.874  0.126 | 262  262 | 0.232  -0.232 | 0.817  0.817 |
| *FCGR3A* p.Val176Phe  -  V  F  VV  FF  VF | 0.006  0.338  0.633  0.005  0.011  0.006 | 19  484  487  14  34  19 | -1.147  -0.277  0.668  0.535  0.169  -2.065 | 0.2513  0.7818  0.5039  0.5930  0.8658  0.0389 |
| *FCGR2B* promoter  2B.1  2B.2  2B.4 | 0.895  0.006  0.099 | 242  18  226 | -0.551  -1.886  1.077 | 0.5818  0.0593  0.2813 |
| *FCGR2C* promoter  -  2B.1  2B.2  2B.1-2B.1  2B.1-2B.2 | 0.047  0.761  0.105  0.079  0.004 | 122  422  233  182  11 | -0.616  0.443  1.181  -1.706  -0.302 | 0.5377  0.6579  0.2378  0.0881  0.7630 |
| *FCGR3B* NA1/NA2/SH  -  NA1  NA2  SH  NA2-NA2  NA1-NA2  NA1-SH | 0.044  0.358  0.526  0.007  0.003  0.05  0.007 | 111  461  483  19  10  112  22 | 0.092  0.289  -0.04  -0.447  -1.265  -0.273  -0.853 | 0.9263  0.7726  0.9679  0.6547  0.2059  0.7851  0.3938 |
| *FCGR2C* ORF/NC ORF/Stop  -  Stop  ORF  NC-ORF  Stop-Stop  NC-ORF-NC-ORF | 0.048  0.75  0.107  0.009  0.07  0.007 | 124  428  235  28  157  20 | -0.612  0.657  1.364  -1.89  -1.372  -0.447 | 0.5408  0.5111  0.1724  0.0588  0.1699  0.6547 |

**Supplementary Table 3**. IVIg case-control analysis in the Australian and United States combined cohort of European descent. KD patients with adequate IVIg responses are compared with patients who were resistant to IVIg therapy. This was done for all the measured CNV and SNPs at the *FCGR2/3* locus. Fisher’s Exact test and single logistic regression was performed on allele frequency of the indicated allele.

| **Variant** | **Allele frequency** | | **Fisher** | **Single logistic regression** | |
| --- | --- | --- | --- | --- | --- |
|  | **IVIg resistant n=56** | **IVIg responsive n=224** | ***P* value** | **OR (LL95-UL95)** | ***P* value** |
| CNR1 ≥ 2 copies  CNR1 < 2 copies | 0.93  0.07 | 0.94  0.06 | 0.76 | 0.86 (0.27-2.72) | 0.80 |
| CNR1 ≤ 2 copies  CNR1 > 2 copies | 0.91  0.09 | 0.92  0.08 | 0.78 | 0.83 (0.29-2.35) | 0.73 |
| CNR2 ≥ 2 copies  CNR2 < 2 copies | 1.00  0.00 | 0.98  0.02 | 0.59 | Inf (0.00-Inf) | 0.98 |
| CNR2 ≤ 2 copies  CNR2 > 2 copies | 0.93  0.07 | 0.95  0.05 | 0.75 | 0.79 (0.25-2.53) | 0.70 |
| CNR3 ≥ 2 copies  CNR3 < 2 copies | 1.00  0.00 | 1.00  0.00 | NA | NA | NA |
| *FCGR2A* p.166His  0 copies  1 copy  2 copies | 0.16  0.48  0.36 | 0.19  0.53  0.28 | 0.49 | 0.78 (0.51-1.21) | 0.27 |
| *FCGR2A* p.62Trp  0 copies  1 copy  2 copies | 0.66  0.32  0.02 | 0.71  0.27  0.02 | 0.64 | 0.81 (0.46-1.42) | 0.45 |
| *FCGR2B* p.232Thr  0 copies  1 copy  2 copies | 0.77  0.23  0.00 | 0.80  0.19  0.01 | 0.73 | 0.90 (0.46-1.76) | 0.76 |
| *FCGR3A* p.176Val  0 copies  1 copy  2 copies  3 copies | 0.38  0.52  0.11  0.00 | 0.39  0.50  0.09  0.01 | 0.94 | 0.97 (0.62-1.50) | 0.88 |
| *FCGR2B* promoter  0 2B.4  1 2B.4  2 2B.4 | 0.71  0.29  0.00 | 0.76  0.22  0.02 | 0.42 | 0.90 (0.50-1.63) | 0.73 |
| *FCGR2C* promoter  0 2B.2  1 2B.2  2 2B.2 | 0.73  0.27  0.00 | 0.72  0.26  0.02 | 0.79 | 1.15 (0.62-2.12) | 0.65 |
| *FCGR3B* NA1/NA2/SH  0 NA1  1 NA1  2 NA1  3 NA1  0 NA2  1 NA2  2 NA2  3 NA2  0 SH  1 SH | 0.07  0.50  0.43  0.00  0.41  0.52  0.07  0.00  0.95  0.05 | 0.10  0.46  0.44  0.00  0.45  0.45  0.10  0.00  0.95  0.05 | 0.85  0.71  1.00 | 0.98 (0.62-1.54)  1.01 (0.65-1.57)  0.99 (0.27-3.64) | 0.94  0.98  0.99 |
| *FCGR2C* ORF/NC-ORF/Stop  0 ORF  1 ORF  2 ORF  3 ORF  0 NC-ORF  1 NC-ORF  2 NC-ORF  0 Stop  1 Stop  2 Stop  3 Stop | 0.70  0.30  0.00  0.00  0.88  0.07  0.05  0.02  0.41  0.52  0.05 | 0.71  0.26  0.02  0.00  0.95  0.02  0.03  0.05  0.34  0.51  0.10 | 0.70  0.07  0.61 | 1.06 (0.60-1.87)  0.58 (0.31-1.08)  1.12 (0.73-1.74) | 0.85  0.09  0.60 |

**Supplementary Table 4**. IVIg case-control analysis in the Dutch cohort of European descent. KD patients with adequate IVIg responses are compared with patients who were resistant to IVIg therapy. This was done for all the measured CNV and SNPs at the *FCGR2/3* locus. Fisher’s Exact test and single logistic regression was performed on allele frequency of the indicated allele.

| **Variant** | **Allele frequency** | | **Fisher** | **Single logistic regression** | |
| --- | --- | --- | --- | --- | --- |
|  | **IVIg resistant n=71** | **IVIg responsive n=198** | ***P* value** | **OR (LL95-UL95)** | ***P* value** |
| CNR1 ≥ 2 copies  CNR1 < 2 copies | 0.92  0.08 | 0.92  0.08 | 1.00 | 0.89 (0.33-2.39) | 0.81 |
| CNR1 ≤ 2 copies  CNR1 > 2 copies | 0.97  0.03 | 0.92  0.08 | 0.42 | 2.83 (0.63-12.69) | 0.17 |
| CNR2 ≥ 2 copies  CNR2 < 2 copies | 0.97  0.03 | 1.00  0.00 | 0.07 | 0.00 (0.00-Inf) | 0.99 |
| CNR2 ≤ 2 copies  CNR2 > 2 copies | 0.93  0.07 | 0.94  0.06 | 0.78 | 0.78 (0.26-2.32) | 0.65 |
| CNR3 ≥ 2 copies  CNR3 < 2 copies | 1.00  0.00 | 1.00  0.00 | NA | NA | NA |
| *FCGR2A* p.166His  0 copies  1 copy  2 copies | 0.17  0.52  0.31 | 0.16  0.51  0.33 | 0.98 | 1.07 (0.72-1.59) | 0.74 |
| *FCGR2A* p.62Trp  0 copies  1 copy  2 copies | 0.75  0.21  0.04 | 0.72  0.27  0.01 | 0.10 | 0.97 (0.56-1.67) | 0.91 |
| *FCGR2B* p.232Thr  0 copies  1 copy  2 copies | 0.72  0.28  0.00 | 0.82  0.17  0.02 | 0.12 | 0.66 (0.37-1.19) | 0.17 |
| *FCGR3A* p.176Val  0 copies  1 copy  2 copies  3 copies | 0.37  0.48  0.14  0.01 | 0.37  0.49  0.13  0.01 | 0.98 | 0.92 (0.62-1.36) | 0.68 |
| *FCGR2B* promoter  0 2B.4  1 2B.4  2 2B.4 | 0.76  0.23  0.01 | 0.74  0.24  0.02 | 0.95 | 1.11 (0.63-1.97) | 0.72 |
| *FCGR2C* promoter  0 2B.2  1 2B.2  2 2B.2 | 0.69  0.27  0.04 | 0.72  0.26  0.02 | 0.53 | 0.82 (0.49-1.37) | 0.45 |
| *FCGR3B* NA1/NA2/SH  0 NA1  1 NA1  2 NA1  0 NA2  1 NA2  2 NA2  0 SH  1 SH | 0.46  0.44  0.10  0.11  0.46  0.42  0.97  0.03 | 0.41  0.46  0.13  0.14  0.44  0.42  0.98  0.02 | 0.78  0.72  0.38 | 1.22 (0.81-1.83)  0.93 (0.63-1.39)  0.53 (0.09-3.24) | 0.34  0.74  0.49 |
| *FCGR2C* ORF/NC-ORF/Stop  0 ORF  1 ORF  2 ORF  0 NC-ORF  1 NC-ORF  2 NC-ORF  0 Stop  1 Stop  2 Stop  3 Stop  4 Stop | 0.69  0.28  0.03  0.99  0.00  0.01  0.04  0.35  0.56  0.04  0.00 | 0.72  0.25  0.03  0.98  0.01  0.01  0.03  0.31  0.59  0.07  0.01 | 0.82  1.00  0.74 | 0.88 (0.53-1.47)  1.04 (0.31-3.54)  1.32 (0.88-2.01) | 0.62  0.95  0.18 |

**Supplementary Table 5**. IVIg case-control analysis in the Polish cohort of European descent. KD patients with adequate IVIg responses are compared with patients who were resistant to IVIg therapy. This was done for all the measured CNV and SNPs at the *FCGR2/3* locus. Fisher’s Exact test and single logistic regression was performed on allele frequency of the indicated allele.

| **Variant** | **Allele frequency** | | **Fisher** | **Single logistic regression** | |
| --- | --- | --- | --- | --- | --- |
|  | **IVIg resistant n=36** | **IVIg responsive n=66** | ***P* value** | **OR (LL95-UL95)** | ***P* value** |
| CNR1 ≥ 2 copies  CNR1 < 2 copies | 0.94  0.06 | 0.91  0.09 | 0.71 | 1.70 (0.32-8.89) | 0.53 |
| CNR1 ≤ 2 copies  CNR1 > 2 copies | 0.86  0.14 | 0.85  0.15 | 1.00 | 1.11 (0.35-3.53) | 0.86 |
| CNR2 ≥ 2 copies  CNR2 < 2 copies | 0.97  0.03 | 1.00  0.00 | 0.35 | 0.00 (0.00-Inf) | 0.99 |
| CNR2 ≤ 2 copies  CNR2 > 2 copies | 0.92  0.08 | 0.97  0.03 | 0.34 | 0.34 (0.05-2.16) | 0.25 |
| CNR3 ≥ 2 copies  CNR3 < 2 copies | 1.00  0.00 | 1.00  0.00 | NA | NA | NA |
| *FCGR2A* p.166His  0 copies  1 copy  2 copies | 0.19  0.47  0.33 | 0.14  0.56  0.30 | 0.61 | 1.06 (0.58-1.95) | 0.84 |
| *FCGR2A* p.62Trp  0 copies  1 copy  2 copies | 0.72  0.22  0.06 | 0.65  0.30  0.05 | 0.66 | 1.21 (0.59-2.48) | 0.61 |
| *FCGR2B* p.232Thr  0 copies  1 copy  2 copies | 0.69  0.31  0.00 | 0.77  0.23  0.00 | 0.48 | 0.67 (0.27-1.67) | 0.39 |
| *FCGR3A* p.176Val  0 copies  1 copy  2 copies | 0.42  0.36  0.22 | 0.26  0.47  0.27 | 0.27 | 1.46 (0.84-2.53) | 0.18 |
| *FCGR2B* promoter  0 2B.4  1 2B.4 | 0.75  0.25 | 0.74  0.26 | 1.00 | 1.04 (0.41-2.65) | 0.93 |
| *FCGR2C* promoter  0 2B.2  1 2B.2  2 2B.2 | 0.64  0.36  0.00 | 0.70  0.27  0.03 | 0.51 | 0.90 (0.41-1.97) | 0.79 |
| *FCGR3B* NA1/NA2/SH  0 NA1  1 NA1  2 NA1  3 NA1  0 NA2  1 NA2  2 NA2  0 SH  1 SH  2 SH | 0.08  0.42  0.47  0.03  0.44  0.47  0.08  0.97  0.03  0.00 | 0.09  0.48  0.42  0.00  0.35  0.58  0.08  0.89  0.09  0.02 | 0.62  0.60  0.62 | 0.77 (0.41-1.44)  1.27 (0.65-2.51)  3.90 (0.50-30.33) | 0.41  0.48  0.19 |
| *FCGR2C* ORF/NC-ORF/Stop  0 ORF  1 ORF  2 ORF  0 NC-ORF  1 NC-ORF  2 NC-ORF  3 NC-ORF  0 Stop  1 Stop  2 Stop  3 Stop  4 Stop | 0.64  0.36  0.00  0.86  0.11  0.03  0.00  0.03  0.39  0.56  0.00  0.03 | 0.70  0.26  0.05  0.86  0.03  0.09  0.02  0.11  0.35  0.50  0.05  0.00 | 0.30  0.22  0.27 | 0.96 (0.45-2.04)  1.30 (0.63-2.71)  0.78 (0.44-1.39) | 0.91  0.48  0.40 |

**Supplementary Table 6**. Summary of studies included in meta-analysis

| **First author, year** | **Country (ethnicity)** | **Population size** | **Polymorphisms investigated and included in meta-analysis** | **Associations with IVIG** | **Associated with CAA** |
| --- | --- | --- | --- | --- | --- |
| *Taniuchi, 2005* | Japan (Asian) | 65 KD cases vs.  566 healthy controls | Fc*γ*RIIa-166H  Fc*γ*RIIIa-176V  Fc*γ*RIIIb-NA1 | Not investigated | Fc*γ*RIIA-131H |
| *Biezeveld, 2006* | The Netherlands, (European) | 167 KD patients vs. 239 unrelated European controls | Fc*γ*RIIa-166H  Fc*γ*RIIb-232T  Fc*γ*RIIIa-176V  Fc*γ*RIIIb-NA1 | Not investigated | Not significant |
| *Shresta, 2011* | United States (European) | 176 KD cases vs. 369 healthy parental controls | Fc*γ*RIIb-232T  Fc*γ*RIIb c.-120A | Fc*γ*RIIb c.-120A | Not investigated |
| *Chatzikyriakidou, 2015* | Greece (European) | 47 KD cases vs. 50 controls | Fc*γ*RIIa-166H | Not investigated | Not significant |
| *Duan, 2014* | China (Asian) | 428 KD cases vs. 493 sex-matched non-related controls | Fc*γ*RIIa-166H | Not investigated | Not significant |
| Abbreviations: KD = Kawasaki disease, CAA = coronary artery aneurysm | | | | | |

**Supplementary Table 7**. CAA case-control analysis in the Australian and United States combined cohort of European descent. KD patients who developed CAA are compared with patients who did not develop CAA. This was done for all the measured CNV and SNPs at the *FCGR2/3* locus. Fisher’s Exact test and single logistic regression was performed on allele frequency of the indicated allele.

| **Variant** | **Allele frequency** | | **Fisher** | **Single logistic regression** | |
| --- | --- | --- | --- | --- | --- |
|  | **No CAA n=259** | **CAA n=103** | ***P* value** | **OR (LL95-UL95)** | ***P* value** |
| CNR1 ≥ 2 copies  CNR1 < 2 copies | 0.92  0.08 | 0.95  0.05 | 0.37 | 0.58 (0.21-1.58) | 0.28 |
| CNR1 ≤ 2 copies  CNR1 > 2 copies | 0.92  0.08 | 0.91  0.09 | 0.84 | 1.09 (0.49-2.46) | 0.84 |
| CNR2 ≥ 2 copies  CNR2 < 2 copies | 0.99  0.01 | 0.99  0.01 | 1.00 | 0.84 (0.09-8.14) | 0.88 |
| CNR2 ≤ 2 copies  CNR2 > 2 copies | 0.95  0.05 | 0.95  0.05 | 1.00 | 0.97 (0.34-2.78) | 0.95 |
| CNR3 ≥ 2 copies  CNR3 < 2 copies | 1.00  0.00 | 1.00  0.00 | NA | NA | NA |
| *FCGR2A* p.166His  0 copies  1 copy  2 copies | 0.18  0.55  0.27 | 0.20  0.35  0.44 | 0.13 | 1.28 (0.91-1.79) | 0.16 |
| *FCGR2A* p.62Trp  0 copies  1 copy  2 copies | 0.68  0.30  0.02 | 0.71  0.26  0.03 | 0.49 | 0.96 (0.61-1.50) | 0.84 |
| *FCGR2B* p.232Thr  0 copies  1 copy  2 copies | 0.78  0.20  0.02 | 0.81  0.18  0.01 | 0.89 | 0.82 (0.49-1.38) | 0.46 |
| *FCGR3A* p.176Val  0 copies  1 copy  2 copies  3 copies | 0.39  0.51  0.09  0.01 | 0.32  0.53  0.15  0.00 | 0.36 | 1.28 (0.91-1.81) | 0.15 |
| *FCGR2B* promoter  0 2B.4  1 2B.4  2 2B.4 | 0.76  0.22  0.02 | 0.74  0.24  0.02 | 0.76 | 1.14 (0.71-1.83) | 0.58 |
| *FCGR2C* promoter  0 2B.2  1 2B.2  2 2B.2 | 0.70  0.28  0.02 | 0.72  0.27  0.01 | 0.90 | 0.90 (0.57-1.44) | 0.66 |
| *FCGR3B* NA1/NA2/SH  0 NA1  1 NA1  2 NA1  3 NA1  4 NA1  0 NA2  1 NA2  2 NA2  3 NA2  0 SH  1 SH | 0.09  0.47  0.43  0.01  0.004  0.46  0.44  0.10  0.004  0.94  0.06 | 0.07  0.52  0.41  0.00  0.00  0.38  0.55  0.07  0.00  0.95  0.05 | 0.70  0.14  0.80 | 0.93 (0.66-1.32)  1.11 (0.78-1.58)  0.83 (0.29-2.35) | 0.68  0.55  0.73 |
| *FCGR2C* ORF/NC-ORF/Stop  0 ORF  1 ORF  2 ORF  3 ORF  0 NC-ORF  1 NC-ORF  2 NC-ORF  0 Stop  1 Stop  2 Stop  3 Stop  5 Stop | 0.69  0.28  0.02  0.004  0.94  0.03  0.03  0.05  0.35  0.54  0.06  0.004 | 0.70  0.29  0.01  0.00  0.93  0.03  0.04  0.05  0.35  0.50  0.10  0.00 | 0.95  1.00  0.79 | 0.92 (0.59-1.44)  1.06 (0.61-1.85)  1.06 (0.77-1.46) | 0.72  0.83  0.73 |

**Supplementary Table 8**. CAA case-control analysis in the Dutch cohort of European descent. KD patients who developed CAA are compared with patients who didn’t develop CAA. This was done for all the measured CNV and SNPs at the *FCGR2/3* locus. Fisher’s Exact test and single logistic regression was performed on allele frequency of the indicated allele.

| **Variant** | **Allele frequency** | | **Fisher** | **Single logistic regression** | |
| --- | --- | --- | --- | --- | --- |
|  | **No CAA n=291** | **CAA n=65** | ***P* value** | **OR (LL95-UL95)** | ***P* value** |
| CNR1 ≥ 2 copies  CNR1 < 2 copies | 0.92  0.08 | 0.88  0.12 | 0.33 | 1.64 (0.70-3.84) | 0.26 |
| CNR1 ≤ 2 copies  CNR1 > 2 copies | 0.94  0.06 | 0.94  0.06 | 1 | 0.99 (0.33-3.04) | 0.99 |
| CNR2 ≥ 2 copies  CNR2 < 2 copies | 0.99  0.01 | 1.00  0.00 | 1 | 0.00 (0-Inf) | 0.98 |
| CNR2 ≤ 2 copies  CNR2 > 2 copies | 0.95  0.05 | 0.92  0.08 | 0.56 | 1.43 (0.51-4.06) | 0.50 |
| CNR3 ≥ 2 copies  CNR3 < 2 copies | 1.00  0.00 | 1.00  0.00 | NA | NA | NA |
| *FCGR2A* p.166His  0 copies  1 copy  2 copies | 0.20 0.50 0.31 | 0.14 0.48 0.38 | 0.40 | 1.33 (0.90-1.97) | 0.16 |
| *FCGR2A* p.62Trp  0 copies  1 copy  2 copies | 0.75 0.23 0.02 | 0.75 0.20 0.05 | 0.39 | 1.09 (0.64-1.84) | 0.76 |
| *FCGR2B* p.232Thr  0 copies  1 copy  2 copies | 0.81 0.18 0.01 | 0.80 0.20 0.00 | 0.84 | 1.00 (0.53-1.90) | 0.99 |
| *FCGR3A* p.176Val  0 copies  1 copy  2 copies  3 copies | 0.38 0.49 0.13 0.01 | 0.38 0.51 0.09 0.02 | 0.69 | 0.95 (0.64-1.41) | 0.80 |
| *FCGR2B* promoter  0 2B.4  1 2B.4  2 2B.4 | 0.77 0.21 0.02 | 0.74 0.23 0.03 | 0.71 | 1.18 (0.70-2.01) | 0.54 |
| *FCGR2C* promoter  0 2B.2  1 2B.2  2 2B.2 | 0.74 0.23 0.03 | 0.72 0.23 0.05 | 0.71 | 1.13 (0.69-1.89) | 0.63 |
| *FCGR3B* NA1/NA2/SH  0 NA1  1 NA1  2 NA1  0 NA2  1 NA2  2 NA2  0 SH  1 SH | 0.42  0.47  0.11  0.13  0.45  0.42  0.98  0.02 | 0.43  0.40  0.17  0.20  0.40  0.40  1.00  0.00 | 0.35  0.45  0.60 | 1.12 (0.75-1.66)  0.82 (0.56-1.21)  0.00 (0-Inf) | 0.58  0.32  0.99 |
| *FCGR2C* ORF/NC-ORF/Stop  0 ORF  1 ORF  2 ORF  0 NC-ORF  1 NC-ORF  2 NC-ORF  0 Stop  1 Stop  2 Stop  3 Stop  4 Stop | 0.74  0.23  0.03  0.98  0.01  0.01  0.03  0.30  0.60  0.07  0.00 | 0.72  0.23  0.05  1.00  0.00  0.00  0.05  0.31  0.55  0.08  0.02 | 0.67  1.00  0.59 | 1.15 (0.69-1.89)  0.00 (0-Inf)  1.00 (0.67-1.49) | 0.59  0.98  1.00 |

**Supplementary Table 9**. CAA case-control analysis in the Polish cohort of European descent. KD patients who developed CAA are compared with patients who didn’t develop CAA. This was done for all the measured CNV and SNPs at the *FCGR2/3* locus. Fisher’s Exact test and single logistic regression was performed on allele frequency of the indicated allele.

| **Variant** | **Allele frequency** | | **Fisher** | **Single logistic regression** | |
| --- | --- | --- | --- | --- | --- |
|  | **No CAA n=76** | **CAA n=43** | ***P* value** | **OR (LL95-UL95)** | ***P* value** |
| CNR1 ≥ 2 copies  CNR1 < 2 copies | 0.89  0.11 | 1.00  0.00 | **0.05** | 0.00 (0-Inf) | 0.99 |
| CNR1 ≤ 2 copies  CNR1 > 2 copies | 0.89  0.11 | 0.79  0.21 | 0.17 | 2.25 (0.80-6.35) | 0.13 |
| CNR2 ≥ 2 copies  CNR2 < 2 copies | 1.00  0.00 | 0.98  0.02 | 0.36 | Inf (0-Inf) | 0.99 |
| CNR2 ≤ 2 copies  CNR2 > 2 copies | 0.96  0.04 | 0.91  0.09 | 0.25 | 2.50 (0.53-11.72) | 0.25 |
| CNR3 ≥ 2 copies  CNR3 < 2 copies | 1.00  0.00 | 1.00  0.00 | NA | NA | NA |
| *FCGR2A* p.166His  0 copies  1 copy  2 copies | 0.14 0.51 0.34 | 0.14 0.51 0.35 | 1 | 1.03 (0.59-1.80) | 0.93 |
| *FCGR2A* p.62Trp  0 copies  1 copy  2 copies | 0.72 0.24 0.04 | 0.60 0.35 0.05 | 0.37 | 1.48 (0.77-2.85) | 0.24 |
| *FCGR2B* p.232Thr  0 copies  1 copy  2 copies | 0.76 0.24 0.00 | 0.77 0.23 0.00 | 1 | 0.98 (0.40-2.36) | 0.96 |
| *FCGR3A* p.176Val  0 copies  1 copy  2 copies | 0.34 0.39 0.26 | 0.23 0.60 0.16 | 0.09 | 1.02 (0.61-1.71) | 0.95 |
| *FCGR2B* promoter  0 2B.4  1 2B.4 | 0.76 0.24 | 0.77 0.23 | 1 | 0.98 (0.40-2.36) | 0.96 |
| *FCGR2C* promoter  0 2B.2  1 2B.2  2 2B.2 | 0.71 0.28 0.01 | 0.63 0.35 0.02 | 0.54 | 1.43 (0.69-2.95) | 0.34 |
| *FCGR3B* NA1/NA2/SH  0 NA1  1 NA1  2 NA1  3 NA1  0 NA2  1 NA2  2 NA2  0 SH  1 SH  2 SH | 0.09 0.49 0.41 0.01  0.41 0.53 0.07  0.92 0.07 0.01 | 0.14  0.47  0.40  0.00  0.23  0.58  0.19  0.88  0.12  0.00 | 0.85  **0.05**  0.67 | 0.83 (0.47-1.44)  **2.15 (1.15-4.01)**  1.24 (0.41-3.79) | 0.50  **0.02**  0.70 |
| *FCGR2C* ORF/NC-ORF/Stop  0 ORF  1 ORF  2 ORF  0 NC-ORF  1 NC-ORF  2 NC-ORF  3 NC-ORF  0 Stop  1 Stop  2 Stop  3 Stop  4 Stop | 0.71  0.26  0.03  0.87  0.05  0.07  0.01  0.08  0.37  0.53  0.03  0.00 | 0.63  0.35  0.02  0.84  0.05  0.12  0.00  0.07  0.33  0.56  0.02  0.02 | 0.73  0.78  0.82 | 1.33 (0.66-2.68)  1.14 (0.64-2.03)  1.24 (0.72-2.11) | 0.43  0.65  0.44 |

**Supplementary Figure 1**. Meta-analysis of all tested SNPs and haplotypes for association with IVIg. Forest plots showing both the OR for common/fixed effects model and random effects model.


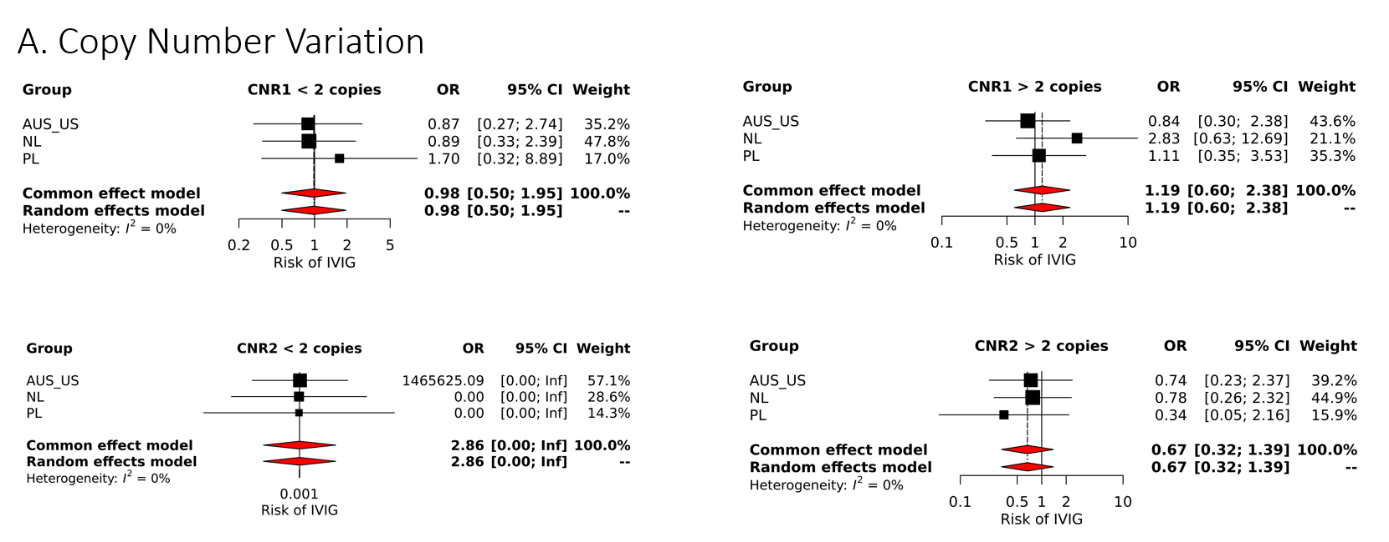

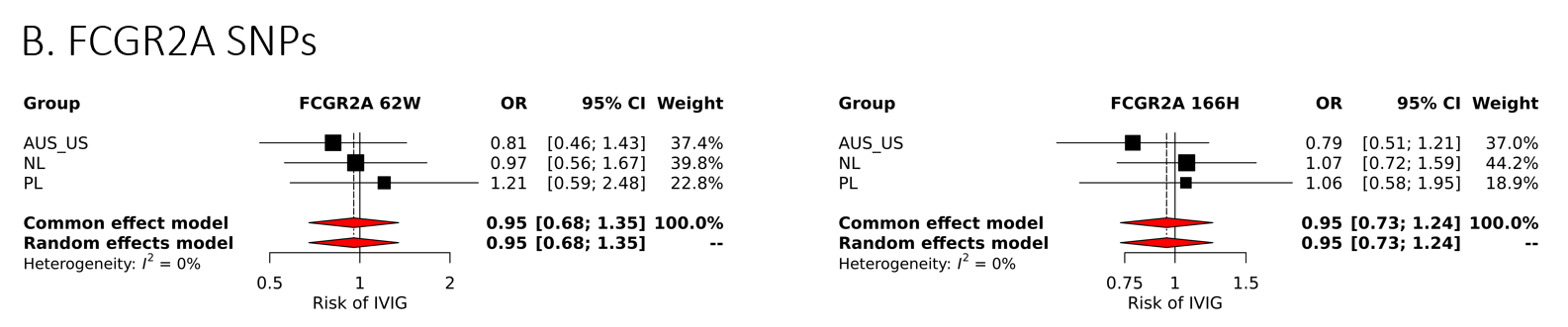

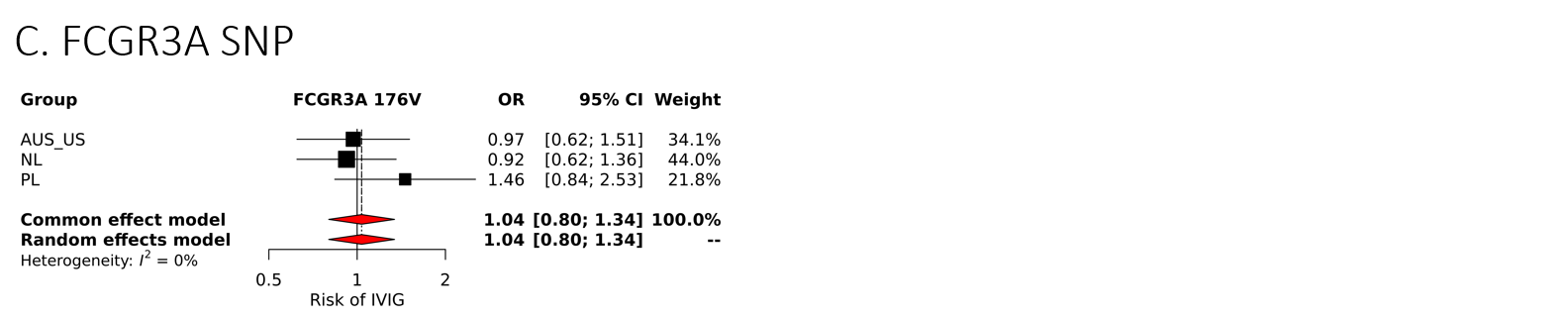

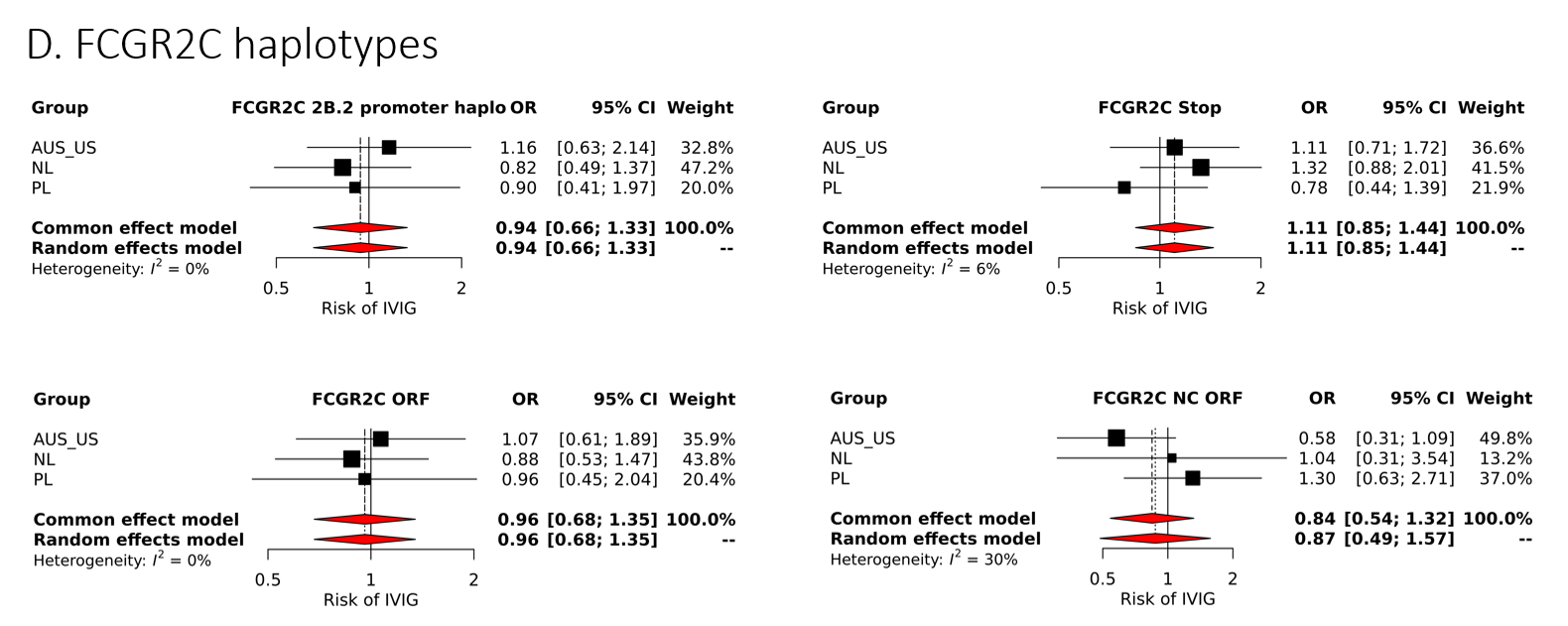

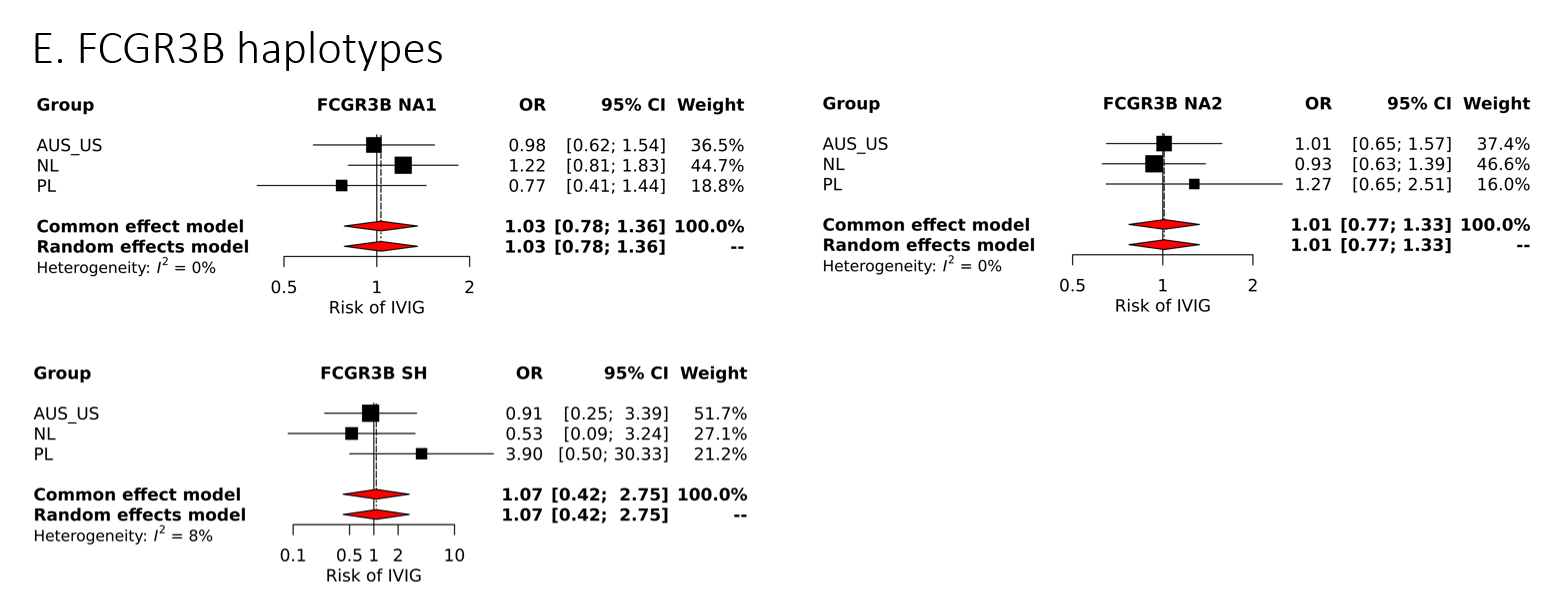

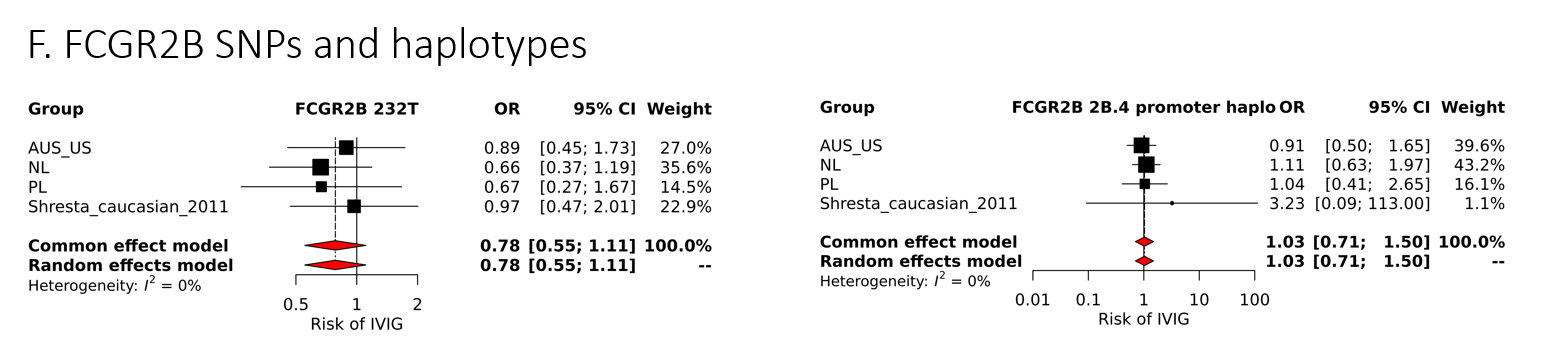


**Supplementary Figure 2**. Identification of studies for the meta-analysis by systematic literature review


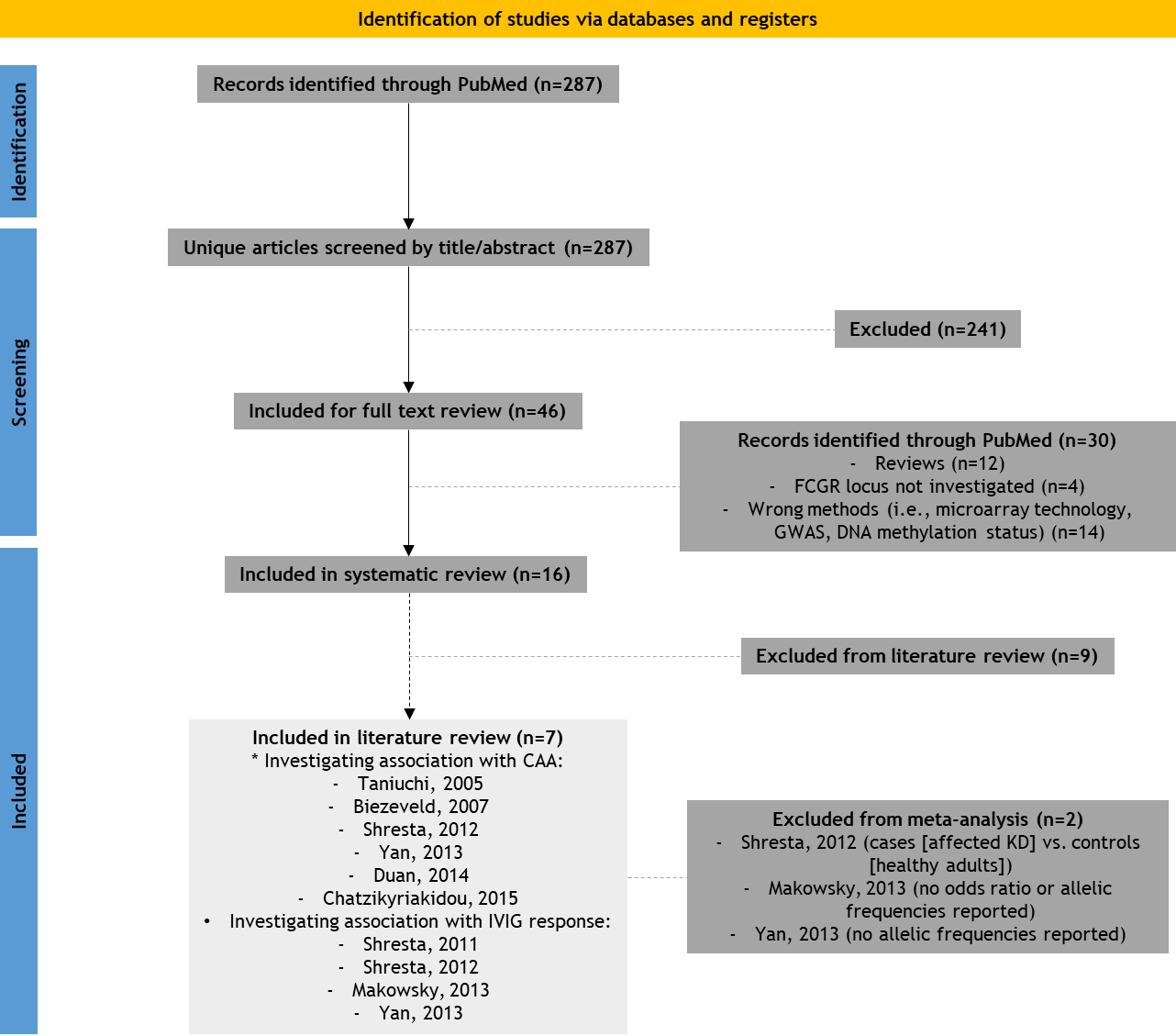


**Supplementary Figure 3**. Meta-analysis of all tested SNPs and haplotypes for association with CAA. Forest plots showing both the OR for common/fixed effects model and random effects model.


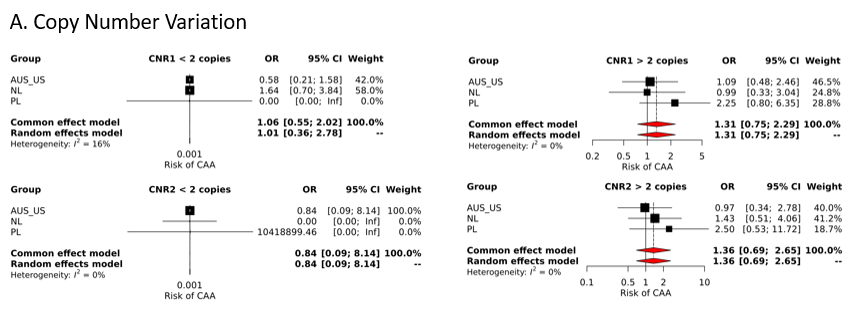


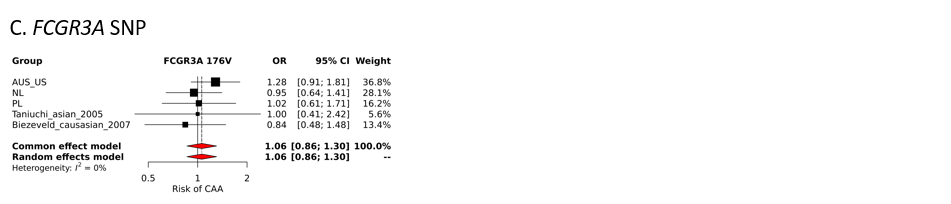

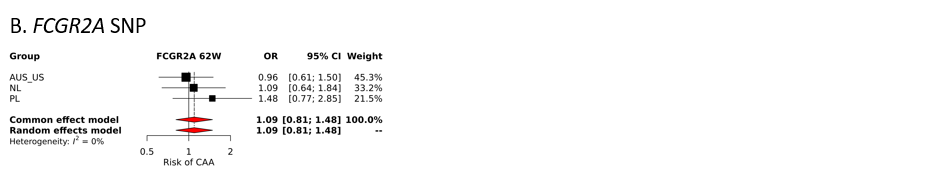


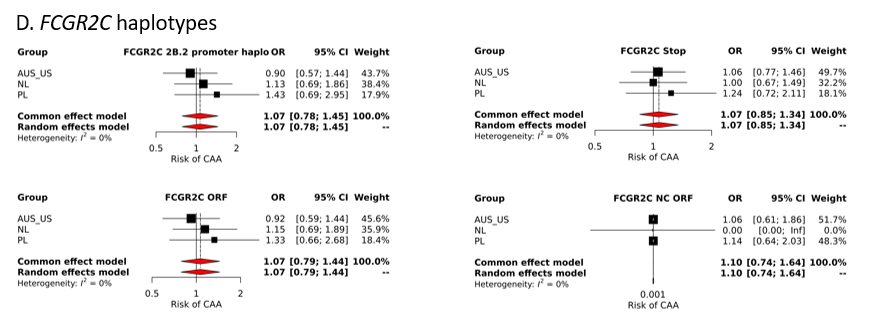


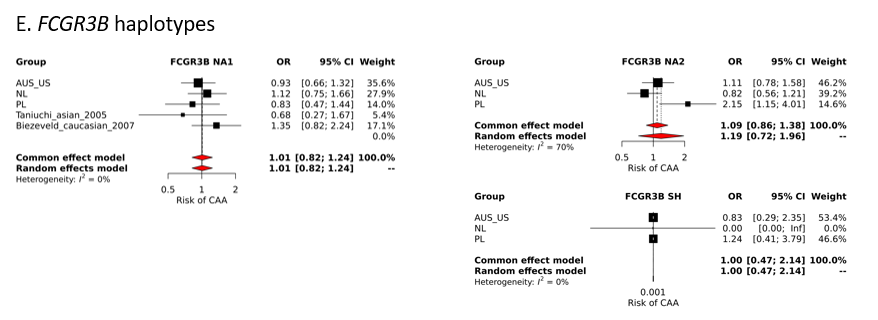


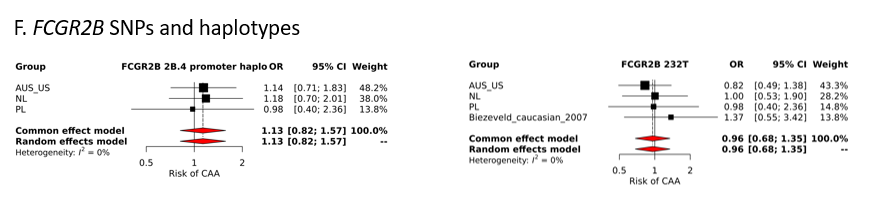

Supplement: Supplementary file 1 [file DataSheet_1.docx]
